# Systematic errors in annotations of truncations, loss-of-function and synonymous variants

**DOI:** 10.3389/fgene.2023.1015017

**Published:** 2023-01-13

**Authors:** Mauno Vihinen

**Affiliations:** Department of Experimental Medical Science, Lund University, Lund, Sweden

**Keywords:** variation annotation errors, protein truncation, loss-of-function variation, synonymous variation, mutation, frameshift variation

## Abstract

Description of genetic phenomena and variations requires exact language and concepts. Vast amounts of variation data are produced with next-generation sequencing pipelines. The obtained variations are automatically annotated, e.g., for their functional consequences. These tools and pipelines, along with systematic nomenclature, mainly work well, but there are still some problems in nomenclature, organization of some databases, misuse of concepts and certain practices. Therefore, systematic errors prevent correct annotation and often preclude further analysis of certain variation types. Problems and solutions are described for presumed protein truncations, variants that are claimed to be of loss-of-function based on the type of variation, and synonymous variants that are not synonymous and lead to sequence changes or to missing protein.

## 1 Introduction

Naming and definition of concepts and things forms the basis of scientific communication. Common language is the foundation; however, difficult to obtain without ambiguity. People often use problematic and sometimes even wrong names for many concepts in genetics. Confusion appears also when people mean different concepts with the same term. The reader cannot always interpret the meaning from the context and can misunderstand the message.

Many types of scientific systematics have been developed since the *Systema Naturae* for taxonomy of species was introduced by Carl von Linné between 1735 and 1768. Some problems in genetic nomenclature and practices have been described earlier (Vihinen, 2015a; Vihinen, 2022a). Here systematic errors in certain types of annotations are discussed, reasons for the problems are charted and remedies suggested.

Next-generation sequencing (NGS) data generation and analysis pipelines produce ever increasing amounts of variation information. As these pipelines are highly computerized, it is instrumental that the used programs make correct choices and are based on accurate definitions, concepts and ideas. Any errors and mistakes in these steps affect the downstream analyses and largely remain unnoticed. Once the variation calls are obtained, variation annotation is used to describe the types of variations in the data. The annotation step largely dictates the outcome of variant interpretation, since variants are selected for interpretation based on the annotations. Popular annotation tools, nomenclature, some databases and practices in the field generate systematic errors. Therefore, certain variants are not properly characterized, e.g. because they are classified as irrelevant or of low significance and relevance in diseases. Nomenclature, annotation tools and databases mainly do great work, the focus here is in issues that still need attention. The concepts discussed and described here have systematic definitions in Variation Ontology (VariO), which facilitates description of variation types, functions, effects, and mechanisms (Vihinen, 2014).

These problems are harmful for scientific inquiry, for detailed and correct descriptions of variations, and in variation interpretation for different purposes. Naturally, there is also impact on the genetic diagnosis of individuals with such variants, which may lead to delayed or missing diagnosis, mistreatment and other problems, in the extreme case to the death of a patient even when treatment would be available.

## 2 Variation annotation

The goal of the variant annotation is to assign functional information to DNA variants. The most basic and the most common annotations describe how variants change the coding sequences and affect the gene products. The annotation of variations is a crucial step in the generation and use of variation data as the annotations are essential, e.g., in the analysis of disease-related variants. If causative variants are misclassified, they are likely ignored in variation interpretation. Only computational approaches are amenable to annotation of whole genome sequences. Every human haploid genome contains some 3.5 to 4.3 million single nucleotide variants (SNVs) and numerous other types of variants, when compared to the reference sequence (Auton et al., 2015).

Tens of variant annotation methods have been developed and can be grouped as theoretically based and empirically based approaches (Samuels et al., 2022). The most widely used of the tools include ANNOVAR (Wang et al., 2010), Ensembl Variant Effect Predictor (VEP) (McLaren et al., 2016), and SnpEff (Cingolani et al., 2012). An important step in the annotation is mapping of variants to reference sequences. Depending on which tool and which sequence is used, the annotations may differ markedly (McCarthy et al., 2014). Recent comparison of ANNOVAR and SnpEff indicated concordance of about 85% when using the same reference sequences (Park and Park, 2021). Thus, variation annotations contain many differences and errors, details depend on the implementation and the used tool. Note that annotation is a general term for addition of new information to data. Thus, e.g. addition of Human Genome Variation Society (HGVS) descriptions (den Dunnen and Antonarakis, 2001) to variants is also a form of annotation.

## 3 Protein truncations

Proteoforms are protein forms originating from a single gene (Smith and Kelleher, 2013) by more than 10 different mechanisms (Vihinen, 2021a). Natural alternative forms appear in some proteins due to N- or C-terminal truncations. These processes are rather common due to alternative translation initiation and termination and other processes (Sharma et al., 2016; Kaushal and Lee, 2021). Proteolytic cleavage is one of the common post-translational modifications and involved in activation and tight regulation of activities of proteins as insulin and proteases. Proteoforms originate from DNA, RNA and protein level alterations. Protein truncation means shortening of polypeptide chain. Systematic definition in VariO is “shortening of protein sequence from terminus” (VariO:0015). Terminological confusion may occur because some scientists consider missing protein as a truncation.

Premature termination codons (PTCs) emerge due to several types of variations and are rather common among disease-causing variants. Some text books, several articles and dictionaries describe PTCs typically as “a codon that has been converted to the same sequence as a stop codon by a non-sense mutation. It is different from a stop codon in that it occurs abnormally and causes premature termination of protein translation resulting in the production of truncated proteins which may be non-functional” (https://www.xmri.com/resource-center/conditions.html?term=92806). This kind of definition is problematic, because in many instances no protein is produced at all.

The HGVS nomenclature (den Dunnen and Antonarakis, 2001) and tools generating annotations based on the nomenclature (Hart et al., 2015; Freeman et al., 2018; Lefter et al., 2021) describe PTC-introducing variants as a protein sequence with a termination signal. These annotations are interpreted as protein truncations. However, most of these variants are not protein truncations, since many PTC-containing mRNA transcripts are degraded and thereby no protein, full length or truncated, is produced. A non-existent protein cannot be truncated! mRNA quality control mechanisms, mainly non-sense-mediated decay (NMD), recognize PTC-containing transcripts and degrade them (Kurosaki et al., 2019). The outcome of a genetic variant depends in addition to the type of the variation also on its context.

NMD machinery recognizes and degrades PTC-containing mRNA transcripts unless the transcript can escape from degradation. NMD escape happens in certain exons. One such scenario is in the penultimate exon within the last 50 nucleotides, called the 50 nt rule (Nagy and Maquat, 1998). Another rule is for the last exon, where variants do not lead to transcript degradation (Le Hir et al., 2001). Recently, additional rules were suggested. Stop codons within 150 nucleotides from the start codon may not cause degradation (Lindeboom et al., 2016). According to the long exon rule, exons longer than 400 bp reduce efficiency of NMD (Lindeboom et al., 2016; Hoek et al., 2019). Numerous other factors affect NMD efficiency and NMD escape, including last exon size, re-initiation of transcription, splice site rescue *etc.*, see (Cummings et al., 2020; Karczewski et al., 2020).

Analysis of about 10,000 matched tumor exomes with the extended rule set suggested that 51% of PTCs trigger efficient NMD (Lindeboom et al., 2019). Additional 27% of PTC variants were predicted to have intermediate NMD effect. Thus, in total 78% of possible PTCs lead at least to partial NMD, which could cause severe effects. This means that 22% of PTCs could indeed be translated and lead to protein truncation. In summary, only about one out of four or five of theoretical truncations in proteins coded by multi-exon transcripts likely have correct annotation.

The degradation of transcripts by NMD means that the coded proteins are not produced. This is a knock-out -type variant and called *missing protein* (VariO:0240) in VariO (Vihinen, 2014). These alterations are among the most common disease-causing variants in many genetic disorders. Missing protein components distort protein complex stoichiometry and can lead to dominant negative phenotype (Veitia and Birchler, 2010). When complex-forming proteins have different abundances, the assembly of the complex is impaired and causes functional consequences.

**FIGURE 1:**
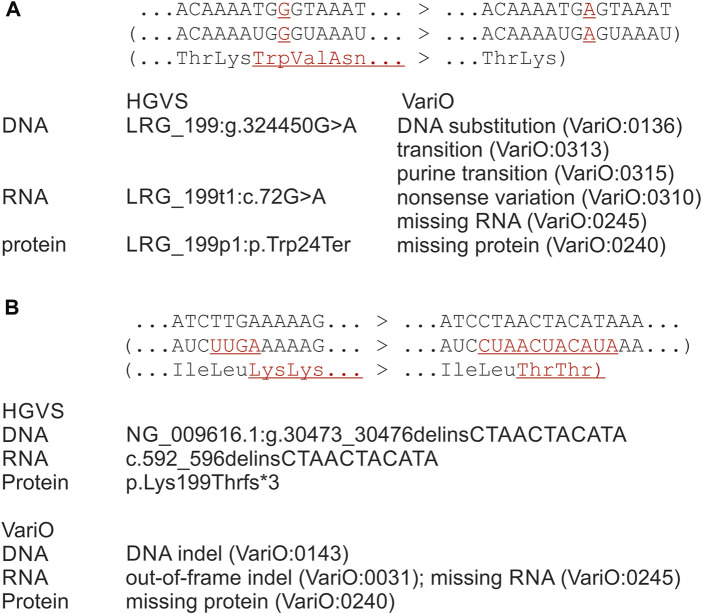
Examples for outcomes of premature stop codon-introducing variants, original sequence to the left and the variant to the right. Systematics both with HGVS and VariO annotations is for observed or predicted outcomes of variants. **(A)** Substitution in *DMD* gene introduces a PTC, therefore the transcript is degraded and no protein in produced. HGVS annotation indicates imaginary effects on RNA and protein levels. Therefore, variants like this are considered as protein truncations, although no protein is produced. VariO annotations of missing RNA and missing protein describe the actual changes. On RNA level, annotation for non-sense variation can be added to indicate what is the reason for missing RNA. **(B)** Indel in *BTK* gene introduces out-of-frame indel at RNA with PTC. No protein is produced due to NMD. The correct HGVS annotations in both examples is on RNA level either r.0 or r (0) and on protein level p.0 or p.(0). HGVS annotations were produced with VariantValidator, which follows the HGVS annotation rules. Differences in the original and variant strands are in red and underlined. RNA and protein changes are in brackets as they are theoretical constructions, in reality the molecules do not exist.

Missing RNA (VariO:0245) and missing protein can be outcomes of several types of variations: substitutions, insertions, deletions, and indels (Vihinen, 2021b). Aberrant splicing that leads to exon skipping or inclusion with consequent reading frame alteration are common PTC-introducing variants (see Figure 1). Aberrant splicing-causing variants can appear either in exon or intron.

The problematic annotations originate from nomenclature, naming tools, annotations and databases. The HGVS nomenclature facilitates the systematic description of variations (den Dunnen and Antonarakis, 2001). It is widely used, and it makes computational searches and analyses possible, however, it still causes also some problems. An example from HGVS nomenclature recommendation for annotation of PTC effect on protein level highlights the issue (Figure 1A): “LRG_199p1:p.Trp24Ter (p.Trp24*) amino acid Trp24 is changed to a stop codon (Ter, *). NOTE: this change is not described as a deletion of the C-terminal end of the protein (i.e. p. Trp24_Met36853del)” (HGVS website http://varnomen.hgvs.org/recommendations/protein/variant/substitution/accesses 2 January 2023).

The isoform one of dystrophin is 3,685 amino acids long and composed of 79 exons. This variant at amino acid 24 is far from the end of the protein, thus the last exon or the 50 nt rules do not apply. The exon is also less than 400 bp long. The site is close to the start codon in exon one and the start-proximal rule might therefore apply. This would mean that there is another functional translation initiation site, which indeed has been seen in certain dystrophin variants (Gurvich et al., 2009). If translation starts from exon 6, the codon for amino acid 24 is not on the translated region at all and cannot thus cause a stop codon. In summary, there is no termination at position 24 as there is no protein expression to be terminated. The HGVS note indicates that it is not annotated as a large C-terminal deletion, however, that is how people interpret the annotation. This protein is either not produced at all due to NMD, or it may be expressed from alternative translation start site; either way, the given protein annotation is incorrect. The correct annotation in the case of NMD is VariO:0240 *missing protein* when using a VariO term and p.(0) in HGVS annotation, in brackets to indicate prediction, or there is another transcript and the N-terminally shorter protein does not contain this amino acid.

The corresponding RNA variation is annotated according to HGVS as LRG_199t1:r.72g>a. In the LOVD DMD database at https://databases.lovd.nl/shared/genes/DMD RNA annotation is r.(?). The transcript is not produced, thus the annotation at RNA should be r.0 or r.(0), or if the alternative transcription initiation site is used based on the new transcript that does not contain this site. The corresponding VariO RNA annotations are VariO:0245 *missing RNA*, or in the new transcript. One could claim there to be an RNA substitution before the RNA is degraded, however, the final outcome is no transcript at all. In the DMD database an unsure RNA annotation r.(?). gives a protein annotation p.(Arg24Ter).

Figure 1B describes another example of PTC-related variation. Indel c.592_595delinsCTAACTACATA in *BTK* gene for Bruton tyrosine kinase (Lougaris et al., 2020) is annotated according to the HGVS nomenclature as p. Lys199Thrfs*3 in protein level. The variant appears in exon eight among 19 exons. Coding region insertions, deletions and indels are either in-frame or out-of-frame, for extensive discussion of RNA variants see (Vihinen, 2021b). For variants to have a chance to be translated the introduced sequence change should be divisible by three to retain coding region and to prevent premature stop. Variants not divisible by three (theoretically two out of three cases) change the coding frame and typically introduce a PTC soon after the variation site. In this example, PTC is introduced in the middle of the transcript and detected by NMD. Therefore, both the RNA and protein annotations provided based on the HGVS nomenclature are incorrect. The correct annotations are r.(0) and p.(0). The PTC-related issues in variation nomenclature are distributed further by computational tools for variation naming, including Mutalyzer (Lefter et al., 2021), Variant Validator (Freeman et al., 2018) and hgvs package (Hart et al., 2015). These methods follow the HGVS nomenclature. Thereby, these problems appear also in variant databases, such as those maintained in LOVD (Fokkema et al., 2021). Variation annotation methods, as ANNOVAR and SnpEff, call such variants as stop gain(ed), but do not describe the outcome of the variants.

If a variant causing missing protein (p.0) is annotated as truncated protein, the users of the annotations may obtain a wrong idea, e.g. about residual activity. Other problems emerge when one wants to understand the causes, mechanisms and effects of these variants and when considering strategies to treat patients. In several neurodegenerative diseases true truncated proteins are mis-localized and cause characteristic disease phenotypes (Jadhav et al., 2013), whereas missing proteins are harmful due to missing activity. NMD activation has been considered as a therapy, e.g., for amyotrophic lateral sclerosis (ALS) to degrade PTC-containing transcripts and to prevent the production of harmful protein forms (Jaffrey and Wilkinson, 2018). Knowledge about whether a protein is truncated or missing can thus have fundamental clinical significance. Thereby, accurate annotation of such variants is of utmost importance.

## 4 Loss-of-function as variation type

Functional consequences of some variations can be called as loss-of-function (lof) or gain-of-function (gof), many variations do not have any functional effect being normal genetic heterogeneity (Vihinen, 2022b). Additional forms of functional effects are antimorphic (antagonistic) and neomorphic (new activity) variants, see (Vihinen, 2021a).

Lof or potential/predicted lof (plof) have been quite widely used as a variant type, e.g., in https://macarthurlab.org/lof/, although it is a functional effect. The practice may simplify terminology, but it generates also confusion and errors. Such predictions may be useful for guiding and prioritizing experimental studies, however, accurate lof and gof statements should always be based on experimental evidence (Vihinen, 2021a), because there is not a clear correlation between variation type and functional effect.

Plof variants have been defined as those with “premature stop (stop-gained), shift-reported transcriptional frame (frameshift), or alter the two essential splice-site nucleotides immediately to the left and right of each exon (splice) found in protein-coding transcripts” (Karczewski et al., 2020). Many such variants indeed cause lof, but not all. Even when there is a lof effect, it may not cause a phenotype. Large numbers of amino acid substitutions have also lof effect, but they have not been included in the (p)lof category.

Plof annotations (Cummings et al., 2020; Karczewski et al., 2020) are produced in an extensive predictive pipeline, which includes numerous rules, analyses and inferences to address different types of variations and various effects. The system has allowed correction of numerous prior errors and generation of a catalogue of plofs, which is very widely used for various purposes, including clinical variant interpretation. The resource is valuable for pinpointing possible lof variants, however, the users should remember that the provided annotations are predictions and are not accurate for all variants. Even when the plof annotation correctly indicates there to be a lof effect, it does not automatically mean that the variant has biological effect that would describe clinical or other consequences of the variant.

As discussed above, more than 20% of PTC variants escape NMD and may thus not be of lof type, for example because of having some residual activity. Similarly, the other types of variants do not have a lof effect in every case. Some splice disruptions, especially those causing exon skipping without RNA frameshift, can code for proteins with some residual activity, which may be sufficient for biological function (Oda et al., 2016; Nakamura et al., 2017). Exon skipping or intronic inclusion can be in-frame in mRNA and code for a protein and have some residual activity. Those variants with out-of-frame alterations typically have an early PTC and are detected by NMD and degraded.

Cells, tissues and organisms have numerous mechanisms that modulate and reduce effects of all kinds of perturbations, including genetic changes. Those mechanisms that reduce effects of variations are called TARAR countermeasures after tolerance, avoidance, repair, attenuation and resistance (Vihinen, 2021a; Vihinen, 2022c). Some of these mechanisms and processes are in-built, intrinsic, while others are active and mounted when there is a perturbation. These mechanisms return the system to sufficient and relevant extent called lagom (Vihinen, 2020a). In the case of the so-called lof variants, tolerance, repair, attenuation and even resistance are relevant. The human body is robust and can tolerate various perturbations, disease tolerance is an example (McCarville and Ayres, 2018). Repair countermeasures, including DNA repair, are highly relevant for phenotypic effects and for correction and rescue of genetic variants (Vihinen, 2022b). Redundant activities are one form of attenuation. Resistance countermeasures actively oppose effects of variants, DNA damage response (Ciccia and Elledge, 2010) is an example of DNA level resistance.

Reading frame errors can be corrected by revertant mosaicism, which restores partly or completely the wild type phenotype or activity by having reversion back to the original sequence (Hirschhorn, 2003). In addition to the actual revertants and other activity-restoring variants within the variant site, variants in other sites may compensate for the alteration, several such variants were seen in T-cell in a single patient (Davis et al., 2010).

Biallelic knock-outs of many human genes do not have a phenotype. Several studies have indicated different numbers of such genes, between 1,285 and 3,230 (MacArthur et al., 2012; Sulem et al., 2015; Lek et al., 2016; Narasimhan et al., 2016; Saleheen et al., 2017). The estimated number of human hemizygous knock-out variants is close to 100 and the number of complete knock-out variants is about 20 in healthy individuals (MacArthur et al., 2012). The knock-outs in these genes could be tolerated because the genes are not needed, at least in the normal living conditions, or there may be redundant activities in ohnologs, countermeasures reduce the effect, *etc.*

Human genome, along with that of many other eukaryotes, is an outcome of two complete genome duplications (Dehal and Boore, 2005), which have produced numerous ohnologs, paralogs, of genes. There are still 7,358 human ohnolog pairs (Singh and Isambert, 2020) and thus a large number of potential redundant activities, which may complement actions of each other and prevent or reduce effects of variants in some cells or situations. Monogenic disease genes are enriched with duplicates and duplicated disease-related genes have higher similarity to their close paralogous genes than other genes (Chen et al., 2013). Redundancy likely masks a substantial number of variants that impair function in a paralog. Moonlighting activities and substrate and catalytic promiscuity of proteins further increase the complexity and reduce the effects of variants (Vihinen, 2021a). Incomplete penetrance is another confounding factor.

When describing lof effects, it is necessary to refer to what function and system and in which level the annotation relates to. A single variant can have different lof effects depending on the level. VariO (Vihinen, 2014) facilitates descriptions of DNA (Vihinen, 2018), RNA (Vihinen, 2021b) and protein (Vihinen, 2015b) variants, consequences, mechanism and functional effects. There are several functions on each of these levels. The DNA functions comprise catalytic activity, information transfer, DNA repair, DNA replication, regulation, reservoir of genetic material, and transcription. The functional effects at the RNA level are amino acid transfer, catalytic activity, regulation, information transfer, splicing function and translation. At the protein level there are seven functions: catalysis, information transfer, movement, recognition, storage, structural protein, and transport activity. Functional effects thus should be described on all the relevant levels and the affected function(s) indicated instead of just claiming variant to be (p)lof. In case there are several transcripts or proteoforms, the functional descriptions should be provided separately for each of them.

A further complication in functional effect discussion is that functional and biological effect may differ. Biological systems are robust and can tolerate even substantial alterations. Several enzymopathies, enzyme-related diseases, display a phenotype only when there is a substantial loss of activity in comparison to normal activity (Vihinen, 2021a). For example, in different forms of hemophilia, 90% or more of the normal activity has to be lost for a patient to display a severe phenotype (Peyvandi et al., 2012; Srivastava et al., 2013). Thus, a variant annotated as categorical (p)lof and indeed having a substantially reduced function may not cause a disease. There are also examples where much smaller activity loss is disease or phenotype-causing (Vihinen, 2021a). In haploinsufficiency, loss of one allele, i.e. 50% of activity, causes the disease in the case of biallelic expression (Morrill and Amon, 2019). It is therefore essential to understand the relevance of the level of activity loss, for extended discussion see (Vihinen, 2021a). For function discussion, the biological function threshold should be defined, but these data are missing for most proteins, even for enzymes, for which the thresholds are among the easiest to define. Therefore, categorical functional annotations cannot apply to all variants and genes/proteins.

The lof annotation issue originates mainly from annotation tools and databases. For example, ANNOVAR (Wang et al., 2010), VEP (McLaren et al., 2016) and Loss-of-Function Transcript Effect Estimator (LOFTEE, https://github.com/konradjk/loftee) assemble variants to the lof category based on the types of variations. Widely used databases Exome Aggregation Consortium (ExAC) database (Karczewski et al., 2017) and Genome Aggregation Database (gnomAD) (Karczewski et al., 2020) are built on the plof principle. Variation type is not a straightforward proxy for functional effects. The models of these tools are too simple to consider the processes, mechanisms, pathways and systems that affect functional effects in living organisms (Vihinen, 2021a).

Similar to lof variants, there are problems also in the use of term gof. It can refer to different types of effects, which are not necessarily clear from the context and the biological impact of which is not clear. Further, the biological relevance of the increased activity is often not known. Many gof and lof effects are without biological consequences and fall within normal biological heterogeneity.

## 5 Synonymous variants

RNA substitutions are typically, and wrongly, divided into three categories: missense, non-sense and synonymous. Note that these terms are relevant only for mRNA sequences, not for DNA or protein variants, see (Vihinen, 2015a). Use of the three categories completely misses the fourth class where variants may look from surface as synonymous, but which in fact affect protein sequence, typically causing missing protein due to NMD degradation of PTC-containing mRNA transcripts (Vihinen, 2022a).

When substitutions are looked only from the perspective of genetic code, many harmful variants are misclassified as synonymous. Even when the variant is synonymous, it can affect DNA, RNA or protein and have non-synonymous effects (Vihinen, 2022a). In DNA, such variants can alter transcription factor binding sites. Even when the coded protein sequence is unaltered, protein activity, structure or regulation may be altered due to a synonymous mRNA change (Sauna and Kimchi-Sarfaty, 2011; Shabalina et al., 2013).

Several apparently synonymous variations are in fact non-synonymous. To facilitate annotation of such variants they were recently renamed as unsense variants (Vihinen, 2022a). The reason why they are not synonymous, although the coding frame looks intact, is that they affect mRNA splicing or splicing regulation or impair regulatory miRNA binding and thus affect protein abundance, often leading to missing protein because of NMD. If looking only at the codon table, these variants look like synonymous. Exonic splice site (Wehr et al., 2018), ESS and ESE (Tonin et al., 2019) variants can impair splicing, lead to various effects and alter the sequence, frequently introducing a PTC. miRNA binding site variants also in the coding region can affect regulation or gene expression and even prevent protein production (Tay et al., 2008). All these variants are systematically ignored by current practice and thus provide wrong annotations.

Unsense variation is described as VariO:0514 “Substitution in mRNA coding region that affects gene expression, protein or protein production without introducing a stop codon in the variation site”. Since unsense variants are currently not annotated, they are largely ignored in clinical variation interpretation and considered irrelevant or of low importance. In reality, these variants have substantial effects and are related to numerous diseases. Causes for these misclassifications are lack of awareness, missing terminology in systematics, and errors in annotation. Therefore, it is important to bear in mind that apparent synonymous variants may be non-synonymous and have various effects. Variants should be called synonymous only based on experimental evidence or reliable predictions (which are not currently available).

## 6 Getting it right

Three factors are instrumental for correcting the discussed challenges and others not covered in here. First, geneticists and other users of genetic data have to become aware of these issues. Second, systematics such as variation nomenclature has to be updated. Third, computer programs, databases and other information resources have to be improved.

Education and increased awareness are key factors in correcting the errors. Full understanding of variation effects is a demanding task and in addition to genetics, the practitioners have to understand proteins, their functions, interactions, structures, networks *etc.* Currently, the field is genetics dominated, which may partially explain also the problems discussed above. Many of the functional effects of variants manifest at protein level, which may not be that familiar a field for geneticists.

Genetics societies should provide educational material to train new and establishes scientists in use of proper nomenclature including the issues introduced above. Text books should be updated to include the latest developments in variation types and effects, as well that in their naming and annotation. In addition, scientific meetings could cover issues listed above and others to increase awareness and to educate the community on correct and accurate practices.

Many systematic classifications relate to variation and variation description. Variation annotation with HGVS nomenclature has to provide terms for all possible variations and outcomes. Relevant terminology has to be introduced (when missing), users trained in correct use of terms and work towards developing exact naming conventions for all kinds of variants. This is already largely in place, but there are still issues to introduce and develop. Practices and pipelines for predictions of functional effects has to be improved and in the mean time warnings could be added to inform users of possible errors and problems. Again, awareness is a key factor. In addition, other systematics relevant for variation data has to be updated, whenever relevant. For example, VariO has ben updated to be consistent.

Even if perfect systematics existed, it is not valuable if not used. Variant annotation and analysis are largely based on computational tools. Therefore, the annotation tools, programs for variant naming, and databases distributing the generated data have to update their routines and pay special attention for currently neglected, ignored and misnamed types of variations.

Variation interpretation for genetic diagnosis in many countries is based on the American College of Medical Genetics and Genomics and the Association of Molecular Pathology (ACMG/AMP) guidelines and standards (Richards et al., 2015). These guidelines have been instrumental in systematizing variation interpretation and in providing more reliable interpretation. The guidelines have also some problems, see, e.g., (Kim et al., 2019; Vihinen, 2020b). I will discuss here how the issues discussed above should be taken into account in variation interpretation according to ACMG/AMP guidelines.

Computational and predictive data, benign supporting BP7: silent variant with non-predicted splice impact. This category relates to many unsense variants. People should pay attention to synonymous variations in exon/intron boundaries but also inside exons as they may not be silent.

PM4, protein length changing variants are currently considered to have moderate support for pathogenic assessment. As many protein truncations are misclassifications, it is important to be careful with such variants. Missing protein instead of truncation is usually a strong evidence for pathogenicity.

PS3, well established functional studies show deleterious effect. The functional studies have to take into account the biological system, since TARAR countermeasures reduce harmful effects of even some substantial change-causing variants. It is not sufficient e.g. to study protein activity just *in vitro*.

BP6 and PP5 are for reputable source of information in other database(s). These may refer e.g. to ExAC, gnomAD and LOVD databases. It is important to consider the quality of these sources and potential problems in them case by case and for different variation types.

For PVS1 criterion, a detailed decision tree has been elaborated for different types of lofs (Abou Tayoun et al., 2018). It takes into account the variation type, possible NMD, splicing effects and other factors. The outcome of the evaluation varies widely from PVS to supporting evidence and there are also cases where annotation cannot be made.

It will never be possible to automatically annotate all variants correctly since many factors contribute to the phenotypes of variants. Therefore, experimental research will always be needed. It should be supported by reliable predictions and annotations that could highlight potential cases for further investigation.

## Data Availability

The original contributions presented in the study are included in the article/supplementary material, further inquiries can be directed to the corresponding author.

## References

[B1] Abou Tayoun A. N. Pesaran T. DiStefano M. T. Oza A. Rehm H. L. Biesecker L. G. (2018). Recommendations for interpreting the loss of function PVS1 ACMG/AMP variant criterion. Hum. Mutat. 39, 1517–1524. 10.1002/humu.23626 30192042 PMC6185798

[B2] Auton A. Brooks L. D. Durbin R. M. Garrison E. P. Kang H. M. Korbel J. O. (2015). A global reference for human genetic variation. Nature 526, 68–74. 10.1038/nature15393 26432245 PMC4750478

[B3] Chen W. H. Zhao X. M. van Noort V. Bork P. (2013). Human monogenic disease genes have frequently functionally redundant paralogs. PLoS Comput. Biol. 9, e1003073. 10.1371/journal.pcbi.1003073 23696728 PMC3656685

[B4] Ciccia A. Elledge S. J. (2010). The DNA damage response: Making it safe to play with knives. Mol. Cell 40, 179–204. 10.1016/j.molcel.2010.09.019 20965415 PMC2988877

[B5] Cingolani P. Platts A. Wang L. L. Coon M. Nguyen T. Wang L. (2012). A program for annotating and predicting the effects of single nucleotide polymorphisms, SnpEff: SNPs in the genome of *Drosophila melanogaster* strain w1118; iso-2; iso-3. Fly. (Austin) 6, 80–92. 10.4161/fly.19695 22728672 PMC3679285

[B6] Cummings B. B. Karczewski K. J. Kosmicki J. A. Seaby E. G. Watts N. A. Singer-Berk M. (2020). Transcript expression-aware annotation improves rare variant interpretation. Nature 581, 452–458. 10.1038/s41586-020-2329-2 32461655 PMC7334198

[B7] Davis B. R. Yan Q. Bui J. H. Felix K. Moratto D. Muul L. M. (2010). Somatic mosaicism in the wiskott-aldrich syndrome: Molecular and functional characterization of genotypic revertants. Clin. Immunol. 135, 72–83. 10.1016/j.clim.2009.12.011 20123155

[B8] Dehal P. Boore J. L. (2005). Two rounds of whole genome duplication in the ancestral vertebrate. PLoS Biol. 3, e314. 10.1371/journal.pbio.0030314 16128622 PMC1197285

[B9] den Dunnen J. T. Antonarakis S. E. (2001). Nomenclature for the description of human sequence variations. Hum. Genet. 109, 121–124. 10.1007/s004390100505 11479744

[B10] Fokkema I. Kroon M. Hernández L. J. A. Asscheman D. Lugtenburg I. Hoogenboom J. (2021). The LOVD3 platform: Efficient genome-wide sharing of genetic variants. Eur. J. Hum. Genet. 29, 1796–1803. 10.1038/s41431-021-00959-x 34521998 PMC8632977

[B11] Freeman P. J. Hart R. K. Gretton L. J. Brookes A. J. Dalgleish R. (2018). VariantValidator: Accurate validation, mapping, and formatting of sequence variation descriptions. Hum. Mutat. 39, 61–68. 10.1002/humu.23348 28967166 PMC5765404

[B12] Gurvich O. L. Maiti B. Weiss R. B. Aggarwal G. Howard M. T. Flanigan K. M. (2009). DMD exon 1 truncating point mutations: Amelioration of phenotype by alternative translation initiation in exon 6. Hum. Mutat. 30, 633–640. 10.1002/humu.20913 19206170 PMC2663021

[B13] Hart R. K. Rico R. Hare E. Garcia J. Westbrook J. Fusaro V. A. (2015). A Python package for parsing, validating, mapping and formatting sequence variants using HGVS nomenclature. Bioinformatics 31, 268–270. 10.1093/bioinformatics/btu630 25273102 PMC4287946

[B14] Hirschhorn R. (2003). *In vivo* reversion to normal of inherited mutations in humans. J. Med. Genet. 40, 721–728. 10.1136/jmg.40.10.721 14569115 PMC1735296

[B15] Hoek T. A. Khuperkar D. Lindeboom R. G. H. Sonneveld S. Verhagen B. M. P. Boersma S. (2019). Single-molecule imaging uncovers rules governing nonsense-mediated mRNA decay. Mol. Cell 75, 324–339. 10.1016/j.molcel.2019.05.008 31155380 PMC6675935

[B16] Jadhav S. Zilka N. Novak M. (2013). Protein truncation as a common denominator of human neurodegenerative foldopathies. Mol. Neurobiol. 48, 516–532. 10.1007/s12035-013-8440-8 23516100

[B17] Jaffrey S. R. Wilkinson M. F. (2018). Nonsense-mediated RNA decay in the brain: Emerging modulator of neural development and disease. Nat. Rev. Neurosci. 19, 715–728. 10.1038/s41583-018-0079-z 30410025 PMC6396682

[B18] Karczewski K. J. Francioli L. C. Tiao G. Cummings B. B. Alföldi J. Wang Q. (2020). The mutational constraint spectrum quantified from variation in 141, 456 humans. Nature 581, 434–443. 10.1038/s41586-020-2308-7 32461654 PMC7334197

[B19] Karczewski K. J. Weisburd B. Thomas B. Solomonson M. Ruderfer D. M. Kavanagh D. (2017). The ExAC browser: Displaying reference data information from over 60 000 exomes. Nucleic Acids Res. 45, D840–D845. 10.1093/nar/gkw971 27899611 PMC5210650

[B20] Kaushal P. Lee C. (2021). N-terminomics - its past and recent advancements. J. Proteomics 233, 104089. 10.1016/j.jprot.2020.104089 33359939

[B21] Kim Y. E. Ki C. S. Jang M. A. (2019). Challenges and considerations in sequence variant interpretation for Mendelian disorders. Ann. Lab. Med. 39, 421–429. 10.3343/alm.2019.39.5.421 31037860 PMC6502951

[B22] Kurosaki T. Popp M. W. Maquat L. E. (2019). Quality and quantity control of gene expression by nonsense-mediated mRNA decay. Nat. Rev. Mol. Cell Biol. 20, 406–420. 10.1038/s41580-019-0126-2 30992545 PMC6855384

[B23] Le Hir H. Gatfield D. Izaurralde E. Moore M. J. (2001). The exon-exon junction complex provides a binding platform for factors involved in mRNA export and nonsense-mediated mRNA decay. Embo J. 20, 4987–4997. 10.1093/emboj/20.17.4987 11532962 PMC125616

[B24] Lefter M. Vis J. K. Vermaat M. den Dunnen J. T. Taschner P. E. M. Laros J. F. J. (2021). Next generation HGVS nomenclature checker. Bioinformatics 37, 2811–2817. 10.1093/bioinformatics/btab051 33538839 PMC8479679

[B25] Lek M. Karczewski K. J. Minikel E. V. Samocha K. E. Banks E. Fennell T. (2016). Analysis of protein-coding genetic variation in 60, 706 humans. Nature 536, 285–291. 10.1038/nature19057 27535533 PMC5018207

[B26] Lindeboom R. G. Supek F. Lehner B. (2016). The rules and impact of nonsense-mediated mRNA decay in human cancers. Nat. Genet. 48, 1112–1118. 10.1038/ng.3664 27618451 PMC5045715

[B27] Lindeboom R. G. H. Vermeulen M. Lehner B. Supek F. (2019). The impact of nonsense-mediated mRNA decay on genetic disease, gene editing and cancer immunotherapy. Nat. Genet. 51, 1645–1651. 10.1038/s41588-019-0517-5 31659324 PMC6858879

[B28] Lougaris V. Soresina A. Baronio M. Montin D. Martino S. Signa S. (2020). Long-term follow-up of 168 patients with X-linked agammaglobulinemia reveals increased morbidity and mortality. J. Allergy Clin. Immunol. 146, 429–437. 10.1016/j.jaci.2020.03.001 32169379

[B29] MacArthur D. G. Balasubramanian S. Frankish A. Huang N. Morris J. Walter K. (2012). A systematic survey of loss-of-function variants in human protein-coding genes. Science 335, 823–828. 10.1126/science.1215040 22344438 PMC3299548

[B30] McCarthy D. J. Humburg P. Kanapin A. Rivas M. A. Gaulton K. Cazier J. B. (2014). Choice of transcripts and software has a large effect on variant annotation. Genome Med. 6, 26. 10.1186/gm543 24944579 PMC4062061

[B31] McCarville J. L. Ayres J. S. (2018). Disease tolerance: Concept and mechanisms. Curr. Opin. Immunol. 50, 88–93. 10.1016/j.coi.2017.12.003 29253642 PMC5884632

[B32] McLaren W. Gil L. Hunt S. E. Riat H. S. Ritchie G. R. Thormann A. (2016). The Ensembl variant effect predictor. Genome Biol. 17, 122. 10.1186/s13059-016-0974-4 27268795 PMC4893825

[B33] Morrill S. A. Amon A. (2019). Why haploinsufficiency persists. Proc. Natl. Acad. Sci. U. S. A. 116, 11866–11871. 10.1073/pnas.1900437116 31142641 PMC6575174

[B34] Nagy E. Maquat L. E. (1998). A rule for termination-codon position within intron-containing genes: When nonsense affects RNA abundance. Trends Biochem. Sci. 23, 198–199. 10.1016/s0968-0004(98)01208-0 9644970

[B35] Nakamura A. Shiba N. Miyazaki D. Nishizawa H. Inaba Y. Fueki N. (2017). Comparison of the phenotypes of patients harboring in-frame deletions starting at exon 45 in the Duchenne muscular dystrophy gene indicates potential for the development of exon skipping therapy. J. Hum. Genet. 62, 459–463. 10.1038/jhg.2016.152 27974813

[B36] Narasimhan V. M. Hunt K. A. Mason D. Baker C. L. Karczewski K. J. Barnes M. R. (2016). Health and population effects of rare gene knockouts in adult humans with related parents. Science 352, 474–477. 10.1126/science.aac8624 26940866 PMC4985238

[B37] Oda H. Sato T. Kunishima S. Nakagawa K. Izawa K. Hiejima E. (2016). Exon skipping causes atypical phenotypes associated with a loss-of-function mutation in FLNA by restoring its protein function. Eur. J. Hum. Genet. 24, 408–414. 10.1038/ejhg.2015.119 26059841 PMC4755370

[B38] Park K. J. Park J. H. (2021). Variations in nomenclature of clinical variants between annotation tools. Lab. Med. 53, 242–245. 10.1093/labmed/lmab074 34612497

[B39] Peyvandi F. Di Michele D. Bolton-Maggs P. H. Lee C. A. Tripodi A. Srivastava A. (2012). Classification of rare bleeding disorders (RBDs) based on the association between coagulant factor activity and clinical bleeding severity. J. Thromb. Haemost. 10, 1938–1943. 10.1111/j.1538-7836.2012.04844.x 22943259

[B40] Richards S. Aziz N. Bale S. Bick D. Das S. Gastier-Foster J. (2015). Standards and guidelines for the interpretation of sequence variants: A joint consensus recommendation of the American College of medical genetics and Genomics and the association for molecular Pathology. Genet. Med. 17, 405–424. 10.1038/gim.2015.30 25741868 PMC4544753

[B41] Saleheen D. Natarajan P. Armean I. M. Zhao W. Rasheed A. Khetarpal S. A. (2017). Human knockouts and phenotypic analysis in a cohort with a high rate of consanguinity. Nature 544, 235–239. 10.1038/nature22034 28406212 PMC5600291

[B42] Samuels D. C. Yu H. Guo Y. (2022). Is it time to reassess variant annotation? Trends Genet. 38, 521–523. 10.1016/j.tig.2022.02.002 35232614

[B43] Sauna Z. E. Kimchi-Sarfaty C. (2011). Understanding the contribution of synonymous mutations to human disease. Nat. Rev. Genet. 12, 683–691. 10.1038/nrg3051 21878961

[B44] Shabalina S. A. Spiridonov N. A. Kashina A. (2013). Sounds of silence: Synonymous nucleotides as a key to biological regulation and complexity. Nucleic Acids Res. 41, 2073–2094. 10.1093/nar/gks1205 23293005 PMC3575835

[B45] Sharma S. Toledo O. Hedden M. Lyon K. F. Brooks S. B. David R. P. (2016). The functional human C-terminome. PLoS One 11, e0152731. 10.1371/journal.pone.0152731 27050421 PMC4822787

[B46] Singh P. P. Isambert H. (2020). OHNOLOGS v2: A comprehensive resource for the genes retained from whole genome duplication in vertebrates. Nucleic Acids Res. 48, D724–D730. 10.1093/nar/gkz909 31612943 PMC7145513

[B47] Smith L. M. Kelleher N. L. (2013). Proteoform: A single term describing protein complexity. Nat. Methods 10, 186–187. 10.1038/nmeth.2369 23443629 PMC4114032

[B48] Srivastava A. Brewer A. K. Mauser-Bunschoten E. P. Key N. S. Kitchen S. Llinas A. (2013). Guidelines for the management of hemophilia. Haemophilia 19, e1–e47. 10.1111/j.1365-2516.2012.02909.x 22776238

[B49] Sulem P. Helgason H. Oddson A. Stefansson H. Gudjonsson S. A. Zink F. (2015). Identification of a large set of rare complete human knockouts. Nat. Genet. 47, 448–452. 10.1038/ng.3243 25807282

[B50] Tay Y. Zhang J. Thomson A. M. Lim B. Rigoutsos I. (2008). MicroRNAs to Nanog, Oct4 and Sox2 coding regions modulate embryonic stem cell differentiation. Nature 455, 1124–1128. 10.1038/nature07299 18806776

[B51] Tonin R. Catarzi S. Caciotti A. Procopio E. Marini C. Guerrini R. (2019). Progressive myoclonus epilepsy in Gaucher Disease due to a new Gly-Gly mutation causing loss of an Exonic Splicing Enhancer. J. Neurol. 266, 92–101. 10.1007/s00415-018-9084-4 30382391 PMC6342868

[B52] Veitia R. A. Birchler J. A. (2010). Dominance and gene dosage balance in health and disease: Why levels matter. J. Pathol. 220, 174–185. 10.1002/path.2623 19827001

[B53] Vihinen M. (2021). Functional effects of protein variants. Biochimie 180, 104–120. 10.1016/j.biochi.2020.10.009 33164889

[B54] Vihinen M. (2022). Individual genetic heterogeneity. Genes (Basel) 13, 1626. 10.3390/genes13091626 36140794 PMC9498725

[B55] Vihinen M. (2015). Muddled genetic terms miss and mess the message. Trends Genet. 31, 423–425. 10.1016/j.tig.2015.05.008 26091961

[B56] Vihinen M. (2022). Generic model for biological regulation. F1000Res. 11, 419. 10.12688/f1000research.110944.1 36128554 PMC9468631

[B57] Vihinen M. (2020). Poikilosis – pervasive biological variation. F1000Res. 9, 602. 10.12688/f1000research.24173.2 32913639 PMC7463298

[B58] Vihinen M. (2020). Problems in variation interpretation guidelines and in their implementation in computational tools. Mol. Genet. Genomic Med. 8, e1206. 10.1002/mgg3.1206 32160417 PMC7507483

[B59] Vihinen M. (2018). Systematics for types and effects of DNA variations. BMC Genomics 19, 974. 10.1186/s12864-018-5262-0 30591019 PMC6309100

[B60] Vihinen M. (2021). Systematics for types and effects of RNA variations. RNA Biol. 18, 481–498. 10.1080/15476286.2020.1817266 32951567 PMC8096339

[B61] Vihinen M. (2015). Types and effects of protein variations. Hum. Genet. 134, 405–421. 10.1007/s00439-015-1529-6 25616435

[B62] Vihinen M. (2014). Variation Ontology for annotation of variation effects and mechanisms. Genome Res. 24, 356–364. 10.1101/gr.157495.113 24162187 PMC3912426

[B63] Vihinen M. (2022). When a synonymous variant is nonsynonymous. Genes (Basel) 13, 1485. 10.3390/genes13081485 36011397 PMC9408308

[B64] Wang K. Li M. Hakonarson H. (2010). ANNOVAR: Functional annotation of genetic variants from high-throughput sequencing data. Nucleic Acids Res. 38, e164. 10.1093/nar/gkq603 20601685 PMC2938201

[B65] Wehr C. Grotius K. Casadei S. Bleckmann D. Bode S. F. N. Frye B. C. (2018). A novel disease-causing synonymous exonic mutation in GATA2 affecting RNA splicing. Blood 132, 1211–1215. 10.1182/blood-2018-03-837336 30030275 PMC6137559

